# Beyond the burrow: Body condition and sex influence exploratory behavior in desert kangaroo rats (*Dipodomys deserti*)

**DOI:** 10.1242/bio.062164

**Published:** 2025-10-15

**Authors:** Katrina Moore, Anahita Sadrossadat, Zhuoyang Zhang, Charles Huang, Joey Huynh, Craig McGowan, Monica Daley

**Affiliations:** ^1^ University of California, Irvine, Department of Ecology and Evolutionary Biology, CA 92617, USA; ^2^ University of Southern California, Division of Integrative Anatomical Sciences, Los Angeles 90007, USA; ^3^ University of California, Irvine, Biomedical Engineering, Henry Samueli School of Engineering, CA 92617, USA; ^4^ University of California, Irvine, Mechanical and Aerospace Engineering, Henry Samueli School of Engineering, CA 92617, USA

**Keywords:** Open-field test, Kangaroo rat, Behavior, Locomotion

## Abstract

Behavioral variation within a population can be influenced by physical factors such as size, sex, and body condition. This variation may contribute to intraspecific niche breadth by enabling individuals to exploit different niches. To examine how anatomy shapes behavior, we conducted open field tests on desert kangaroo rats (*Dipodomys deserti*, *n*=16) and compared their activity to sex, morphology, and body condition. We constructed an arena within the species' natural habitat to simulate ecologically relevant conditions and recorded behavior over 15 min. We quantified speed and distance traveled, used principal component analysis to explore behavioral patterns, and used linear models to test for associations between behavior, locomotor traits, and anatomical variables. We found that individuals with lower body condition scores spent more time exploring, males were more exploratory than females, and individuals with longer feet – a proxy for skeletal size – traveled further. However, behavior and locomotor performance were not significantly correlated. Lastly, individuals moved faster and farther on full moon nights compared to new moon nights, indicating that moonlight influences movement strategy – potentially reflecting trade-offs between foraging and predation risk. These findings highlight species-specific drivers of behavioral variation and underscore the importance of understanding behavioral variability of desert mammals.

## Teaser

**Summary:** Anatomy and moonlight influence exploratory behavior and movement in desert kangaroo rats, highlighting key factors driving behavioral variation in desert mammals.

## INTRODUCTION

An animal's behavior is central to their survival as it allows them to meet their physical needs and navigate complex environmental pressures. These behaviors are influenced by a variety of intrinsic and extrinsic factors such as anatomy, physiology, and interactions with the environment. For example, hormones can trigger physiological responses that alter behavior, as is seen in gerbils where higher fecal glucocorticoid concentrations lead to increased foraging time and greater food consumption (St. Juliana et al., 2017). Genetics also play a significant role in behavior, as certain inherited traits, such as those influencing the dispersal distance of great tits (*Parus major*) from their natal nest, are strongly correlated with an individual's genetic makeup (Korsten et al., 2013). Additionally, environmental factors affect behavior, as demonstrated by meerkats, which exhibit a significant reduction in cooperative vigilance during drought conditions (Rauber et al., 2019). These examples underscore the dynamic nature of animal behavior and the complex interplay of factors that drive individual variation within populations. While it is necessary to understand behavior at the population-level, a growing body of research emphasizes the importance of studying individual differences, as these provide a more nuanced view of how animals uniquely interact with their environment to ensure survival (Shaw, 2020). This approach highlights the need to consider behavioral variation within populations and how specific behaviors are expressed across different individuals.

Risk-related behavior is shaped by a variety of factors, including species-specific trade-offs, physical traits, and individual variation. In this paper, we use ‘risk’ specifically to refer to the potential for predation or other threats to survival. While risk can be considered in other contexts, such as foraging and competition, our focus follows prior work emphasizing the central role of predation and threat avoidance in shaping animal decision-making (Lima and Dill, 1990). In this context, animals that exhibit greater risk tolerance and exploratory behavior may benefit from increased access to resources, yet also face heightened exposure to predators. These trade-offs vary by species; for instance, prey species may prioritize predator avoidance, leading to reduced exploratory behavior. In sister species of toothcarp (*Brachyrhaphis roseni* and *Brachyrhaphis terrabensis*), *B*. *roseni*, which faces less predation in its natural environment, is more active and exploratory in a novel environment (Money et al., 2017). Physical traits can also contribute to variation in risk-taking. For example, bolder Bishop toothcarp (*Brachyrhaphis episcopi*) have a larger body mass compared to shyer individuals of similar body length, suggesting that bold behavior may enable greater access to resources, resulting in higher energy reserves and fitness (Brown et al., 2007). However, the relationship between body size and risk-taking behavior varies across species. For example, guppies (*Poecilia reticulata*) show sex-specific differences in risk-taking, while body size has no influence on this behavior (Harris et al., 2010). Other species demonstrate more complex patterns; for instance, smaller Iberian rock lizards (*Lacerta monticola*) with larger heads and better body condition display greater boldness (López et al., 2005). These findings illustrate the complex interplay between morphology, behavior, and ecological context in shaping risk sensitivity and highlight the importance of studying individual differences across species to gain a deeper understanding of behavioral adaptation.

Exploratory behavior provides a window into individual risk-taking tendencies, which can be measured through an open field test. This method, adaptable to field settings, facilitates standardized comparisons of behavior across individuals and populations while minimizing stress (Aliperti et al., 2021; Gould et al., 2009). Exploratory behavior and risk aversion are closely linked with less exploratory individuals often exhibiting greater risk aversion, as seen in studies of great tits (Van Oers et al., 2004) and multiple rodent species (Hughes et al., 2024 preprint). Additionally, prey species may further limit their exploratory behavior by using freezing behavior to avoid predator detection (Eilam, 2005). Physical traits can also influence exploratory tendencies. Individuals with higher locomotor capacity, due to larger body size, longer legs or relatively higher muscle mass often explore more. For example, larger corroboree frogs (*Pseudophryne corroboree*) were found to be more exploratory in a novel environment compared to smaller conspecifics (Kelleher et al., 2017) and zebrafish artificially selected for exploratory behavior exhibited larger tails and faster locomotor start performance (Kern et al., 2016) – traits which may primarily enhance escape responses but could also facilitate greater exploration by reducing perceived risk. These findings underscore the interconnectedness of behavioral and physical traits in shaping individual variation in risk-taking and exploration.

Desert kangaroo rats (*Dipodomys deserti*) are naturally exploratory and bold, frequently foraging in the open regardless of food availability or light, likely due to their larger size compared to sympatric rodent species (Kotler, 1984). They play a vital role in the desert ecosystems of southwestern North America, dispersing seeds, shaping the landscape through their extensive burrow systems, and serving as a key food source for desert mammals, raptors, and snakes (Goldingay et al., 1997; Longland and Ostoja, 2013). Notably, *D. deserti* use vertical jumps and kicks to evade rattlesnake predation (Freymiller et al., 2019). While vigilance and rapid escape responses are essential for evading predators, slower exploratory locomotion, during which elastic energy is stored and returned with each hop (Christensen et al., 2022), is crucial for efficient foraging in the open desert. Consequently, there may be a fitness trade-off associated with risk-sensitivity, since risk tolerance benefits food acquisition, but risk sensitivity benefits predator escape. Such fitness trade-offs may result in individual variation in risk sensitivity depending on how physical traits and environmental factors influence the optimal strategy. While *D. deserti* has been extensively studied in laboratory settings, this species tames easily in captivity (McGowan and Collins, 2018), thus necessitating field-based research to obtain a more comprehensive and ecologically relevant understanding of sources of individual behavioral variation. We quantified the exploratory behavior and locomotor performance of wild desert kangaroo rats using an open field test within the animals' natural environment. This approach allows us to examine individual variation in risk-taking propensity and the factors influencing such variation.

Since larger individuals may have longer legs, greater muscle mass and power capacity, we hypothesize that large individuals exhibit more exploratory behaviors and faster speeds when exposed to an open field test. To test this hypothesis, we recorded the behavior of desert kangaroo rats (*n*=16) for 15 min in an enclosed arena placed within or near their home territory. The arena was designed to contain their natural substrate to allow for an ecologically relevant range of locomotor behaviors, and contained a shrub, novel object and food patch hidden in the sand. Our goal was to quantify individual variation in a semi-controlled, semi-natural context characterized by elevated novelty, stress, and perceived risk; accordingly, we intentionally limited acclimation to minimize handling time and exposure to the experimental setting. We tracked ten behaviors and measured locomotor performance to examine correlations between exploration, risk-avoidance behaviors, and locomotor measures. Although each individual was tested only once, precluding an assessment of behavioral consistency across contexts that would be necessary to define personality or a behavioral syndrome (Sih et al., 2004), our study provides valuable initial insights into individual variation in exploratory behavior in a consistent semi-controlled context and establishes a foundation for future behavior and personality research in this species. This work contributes to knowledge of the factors that influence risk response in *D. deserti*, informing understanding of synergistic drivers of animal behavior, and provides a novel approach for minimally invasive studies of animal behavior in ecologically relevant conditions, which could be applied to a variety of species.

## RESULTS

Over the course of seven nights (three nights in 2021 and four nights in 2022), we surveyed approximately 135 burrows across an area of roughly 85 hectares. At each burrow, a trap was placed at every tunnel opening (approximately 1-5 traps per burrow). We evaluated 16 adult individuals (seven females, nine males) that ranged in mass from 55-137 g (mean: 103 g) and had foot lengths from 4.4-5.5 cm (mean: 4.9 cm; Table S1). Individuals varied widely in both the total time spent and number of bouts per behavior. The most frequently observed behaviors were hopping (mean duration±s.d.: 478.7±200.7 s; mean bouts: 22±10.1) and hiding in the shrub (291.5±273.5 s; 4.4±2.5 bouts). Several behaviors occurred with moderate frequency, including resting (86.0±55.3 s; 9.4±5.3 bouts), digging (27.6±59.7 s; 2.8±3.6 bouts), eating (19.3±44.5 s; 0.8±2.0 bouts), grooming (16.7±20.5 s; 1.6±1.9 bouts), standing (11.6±9.5 s; 8.1±6.3 bouts), and interacting with the mirror (8.0±9.8 s; 2.3±2.7 bouts). In contrast, jumping (1.7±2.3 s; 1.4±1.8 bouts) and sandbathing (0.8±2.9 s; 0.1±0.3 bouts) were rare and typically brief in duration (Fig. S1, Table S3). In addition to variation in behavior, individuals also differed in their overall movement patterns, with mean speed averaging 23.2±10.1 cm/s and total distance traveled averaging 93.4±64.2 meters (Figs S2 and S3).

### Principal component analysis

We evaluated the top two principal components, whose scores explained 31.2% and 15.6% of the variance in the dataset, cumulatively accounting for 45.5% of the variance in measured behaviors. We chose to only include the first two principal components (hereafter ‘PC1’ and ‘PC2’) in our subsequent analysis, as each principal component beyond PC2 explained less than 15% of the variance. This decision was further supported by a scree plot, which showed an inflection after PC2. We interpreted the principal component axes based on behaviors with loadings greater than half the absolute value of the highest loading for each principal component (>0.16 for PC1; >0.22 for PC2).

Lower scores of PC1 corresponded to individuals spending more time in the shrub, whereas individuals with higher PC1 exhibited higher number of bouts and more time spent locomoting, jumping, standing, resting, grooming, eating, and interacting with the mirror. Additionally, higher PC1 scores signify a higher number of digging bouts, although not necessarily more total time spent digging (Fig. 2). Thus, we interpret this component to be an indicator of risk-taking propensity with risk-averse individuals scoring lower and risk-tolerant, more exploratory individuals scoring higher in PC1.

**Fig. 1.**
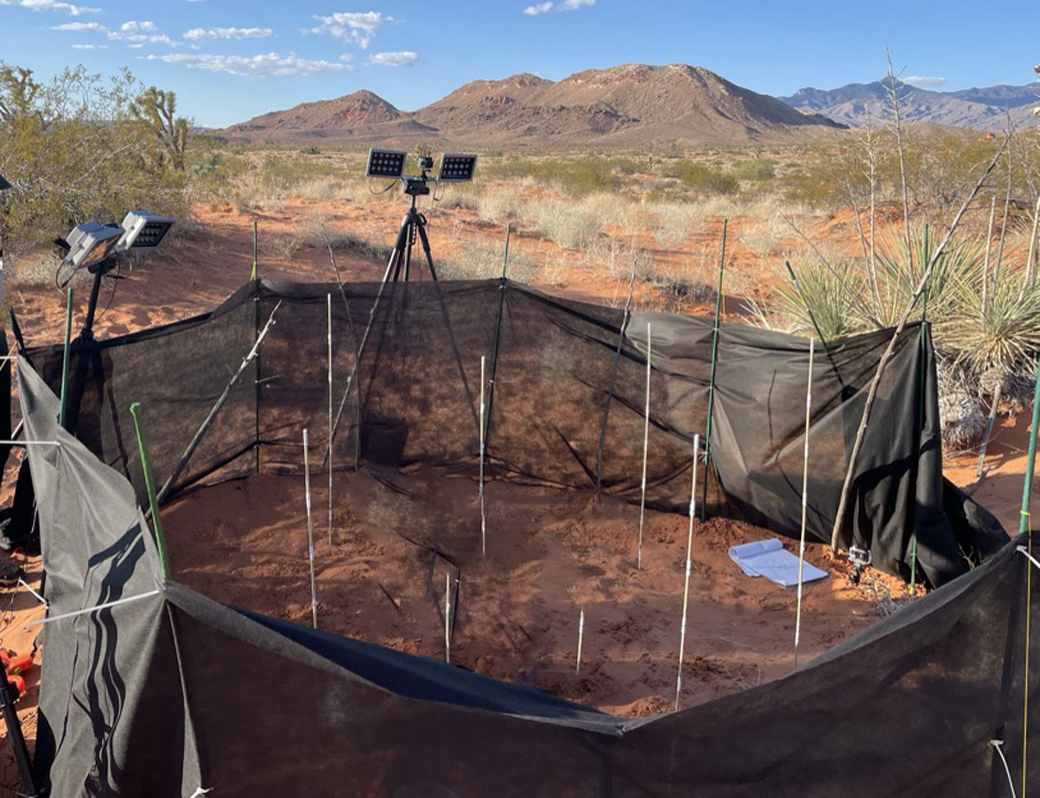
**Image of the behavioral arena set up within a natural environment.** Note that the shrub in the lower right corner is obscured by the arena wall. One wall panel is lowered, as this photograph was taken during daytime arena set up. During active experiments, all wall panels were raised, and the base of the cloth wall was buried to prevent animal escape.

**Fig. 2.**
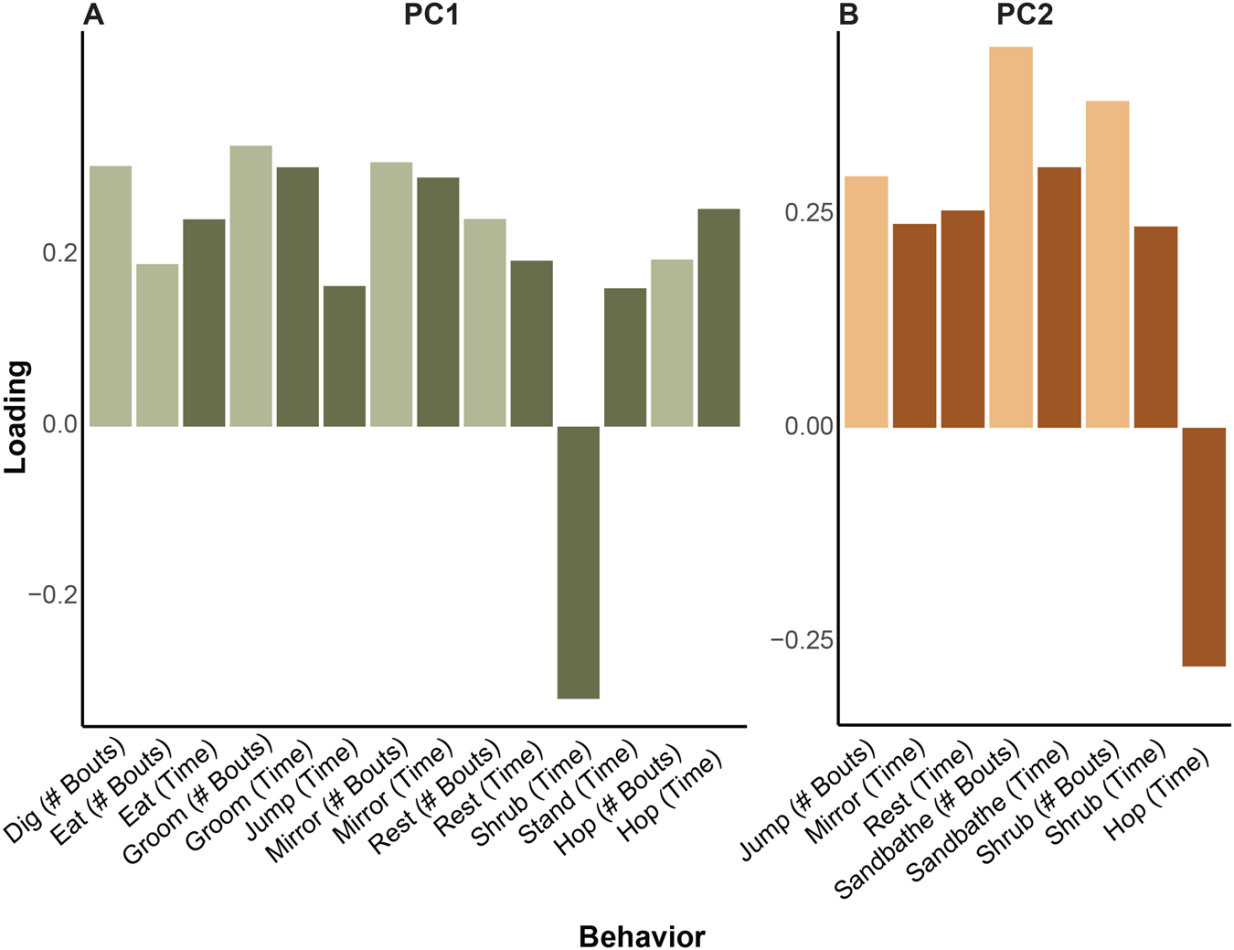
**Bar plot of the highest loading behavior variables for PC1 and PC2 (>0.16 for PC1; >0.22 for PC2).** Dark bars indicate proportional time spent performing a behavior and light bars indicate the number of times (‘bouts’) that behavior was performed.

Individuals with lower PC2 scores spent more time specifically locomoting while those with higher PC2 scores exhibited a larger number of bouts and spent more time in the shrub, resting, sandbathing, interacting with the mirror and jumping, (Fig. 2). Thus, while PC1 differentiated those individuals who mainly hid within the shrub versus all other behaviors, PC2 differentiated variation in how individuals behaved while navigating the open arena. Individuals with low PC2 scores may exhibit more overall ambulatory exploration, whereas those with high PC2 scores tend to display reactive and territorial behaviors and spend less time actively exploring (Fig. 5). Therefore, PC2 may reflect differences in how individuals respond to novel stimuli, such as the unfamiliar enclosure and mirror, ranging from remaining under cover to actively exploring and engaging with novel objects. These patterns could be influenced by perceived risk exposure in this context or by a greater willingness to explore, which may resemble boldness. However, without repeated measures across contexts, we cannot infer personality, and multiple factors likely contributed to the observed variation.

### Linear mixed-effects model

#### Predictors of behavioral principal components (PCs)

##### PC1

**Fig. 3.**
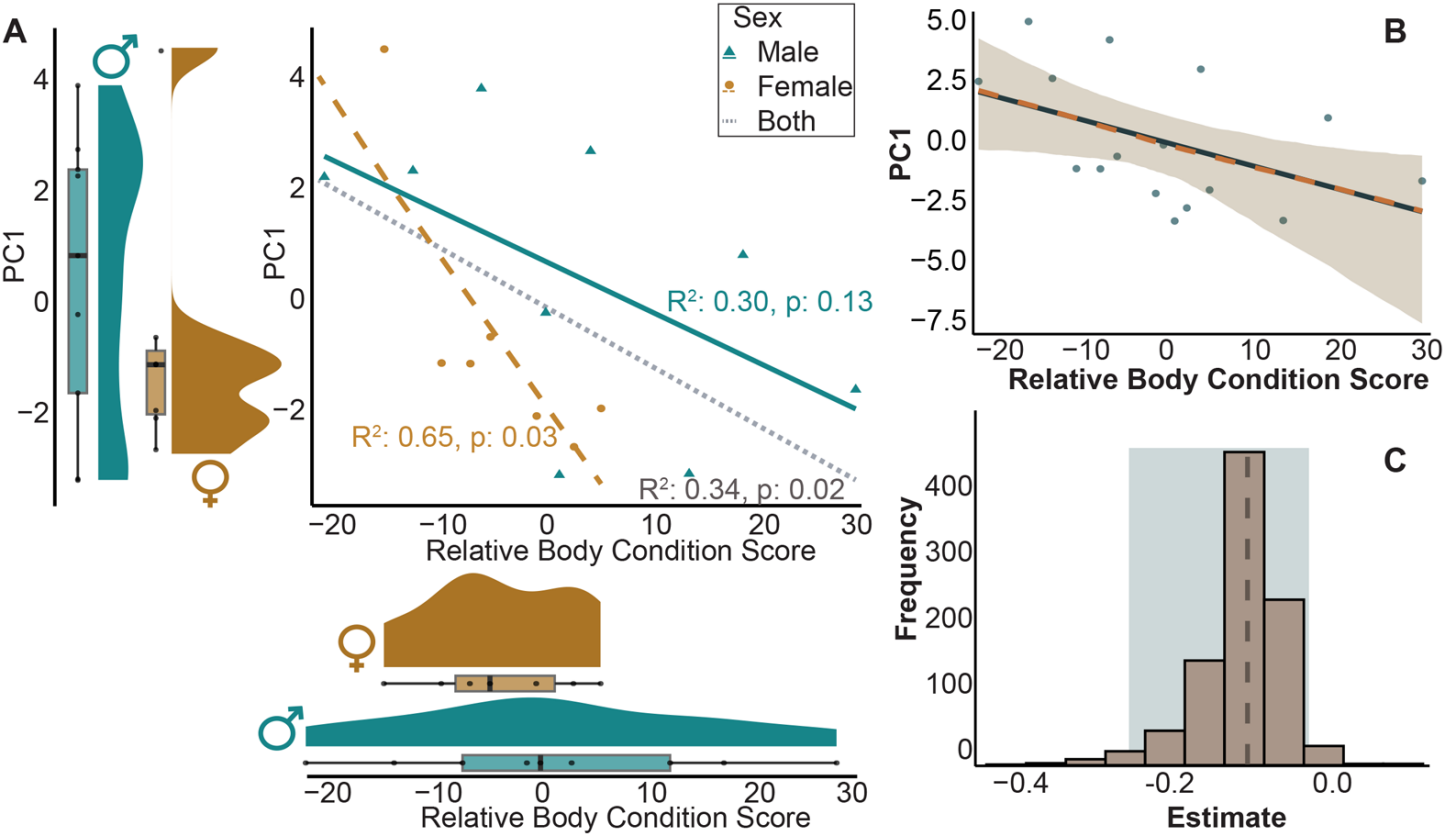
**(A) Linear regression showing relationship between relative body condition score, sex, and PC1.** Males exhibited wider variance in relative body condition scores and PC1 scores when compared to females, which had lower average body conditions and lower average PC1 scores. (B) Bootstrapped regression of PC1 and relative body condition score where the solid line represents the slope of the original model, the dashed line represents the mean of the bootstrap results (lines overlap because slopes are similar), the ribbon indicates the 95% confidence interval, and the points indicate the original data. (C) Bootstrapped estimates (slope) of regression between PC1 and relative body condition score. The dashed line indicates the mean slope, and the shaded box indicates the 95% confidence interval (which does not overlap 0).

The model of best fit for predicting PC1 scores included sex, body condition score and their interaction. Males were more likely to have higher PC1 scores and individuals with higher relative body conditions scored lower in PC1 (Table 1 and Table S4; Fig. 3A).

**Table 1.** Model estimate and ANOVA output for fixed effects from model selection

Response variable	Fixed effect	Estimate	F-statistic	*P*-value
PC1	(Intercept)	−1.77	5.06	**0.04**
	Sex (male)	2.56	6.70	**0.02**
	Body condition	−0.28	7.27	**0.02**
	Sex×body condition	0.19	2.76	0.12
PC2	(Intercept)	−0.99	2.99	0.11
	Body condition	0.09	7.50	**0.02**
	Moon phase (new)	1.76	4.72	**0.05**
Mean speed	(Intercept)	0.33	269.1	**<0.001**
	Moon phase (new)	−0.17	39.5	**<0.001**
Total distance traveled	(Intercept)	−401.24	4.94	**0.04**
	Foot length	108.48	9.03	**0.01**
	Moon phase (new)	−76.22	15.96	**0.002**

The mean slope of the bootstrapped model predicting PC1 based on body condition score was slightly less negative than the slope of the actual model (bootstrapped: −0.10; actual: −0.28). However, the bootstrapped confidence interval (−0.25, −0.02) did not overlap zero, supporting the validity of this correlation (Fig. 3B,C).

##### PC2

The model of best fit for predicting PC2 included relative body condition and moon phase. Individuals with higher body condition scores had higher PC2 scores (*P*=0.02). Although the model suggested that PC2 scores tended to be higher during the new moon compared to full moon (*P*=0.05, Table 1 and Table S4), the spread of data overlapped considerably, and the apparent difference may have been driven by a few outliers. As such, we cannot conclude that moon phase has a true effect on PC2 (Fig. S4).

**Fig. 4.**
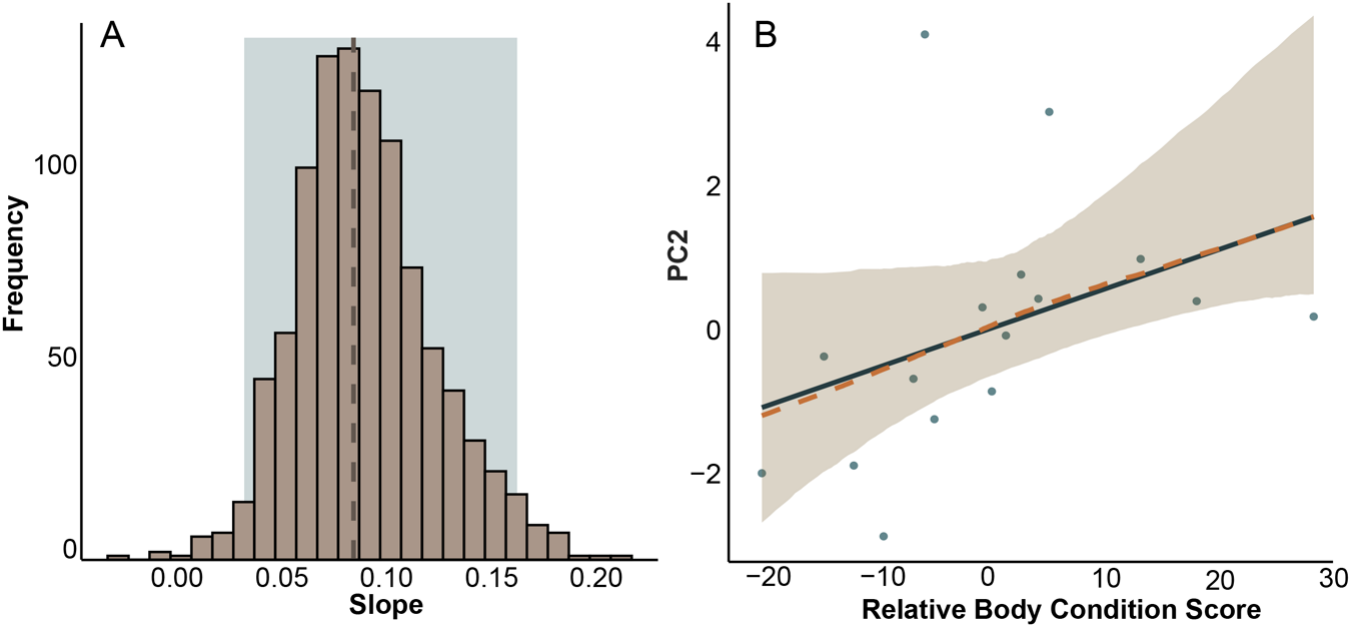
**(A) Bootstrapped estimates (slope) of regression between PC2 and relative body condition score.** The dashed line indicates the mean, and the shaded box indicates the 95% confidence interval. (B) Bootstrapped regression of PC2 and relative body condition score where the solid line represents the slope of the original model (R^2^=0.12), the dashed line represents the mean of the bootstrap results, the ribbon indicates the 95% confidence interval, and the points indicate the original data.

The mean slope of the bootstrapped model (0.1) predicting PC2 based on body condition score was similar to that of the actual model (0.09). The bootstrapped confidence interval (0.04, 0.17) did not overlap zero, indicating that while the effect of body condition score on PC2 may be small, it is unlikely to be due to chance (Fig. 4).

**Fig. 5.**
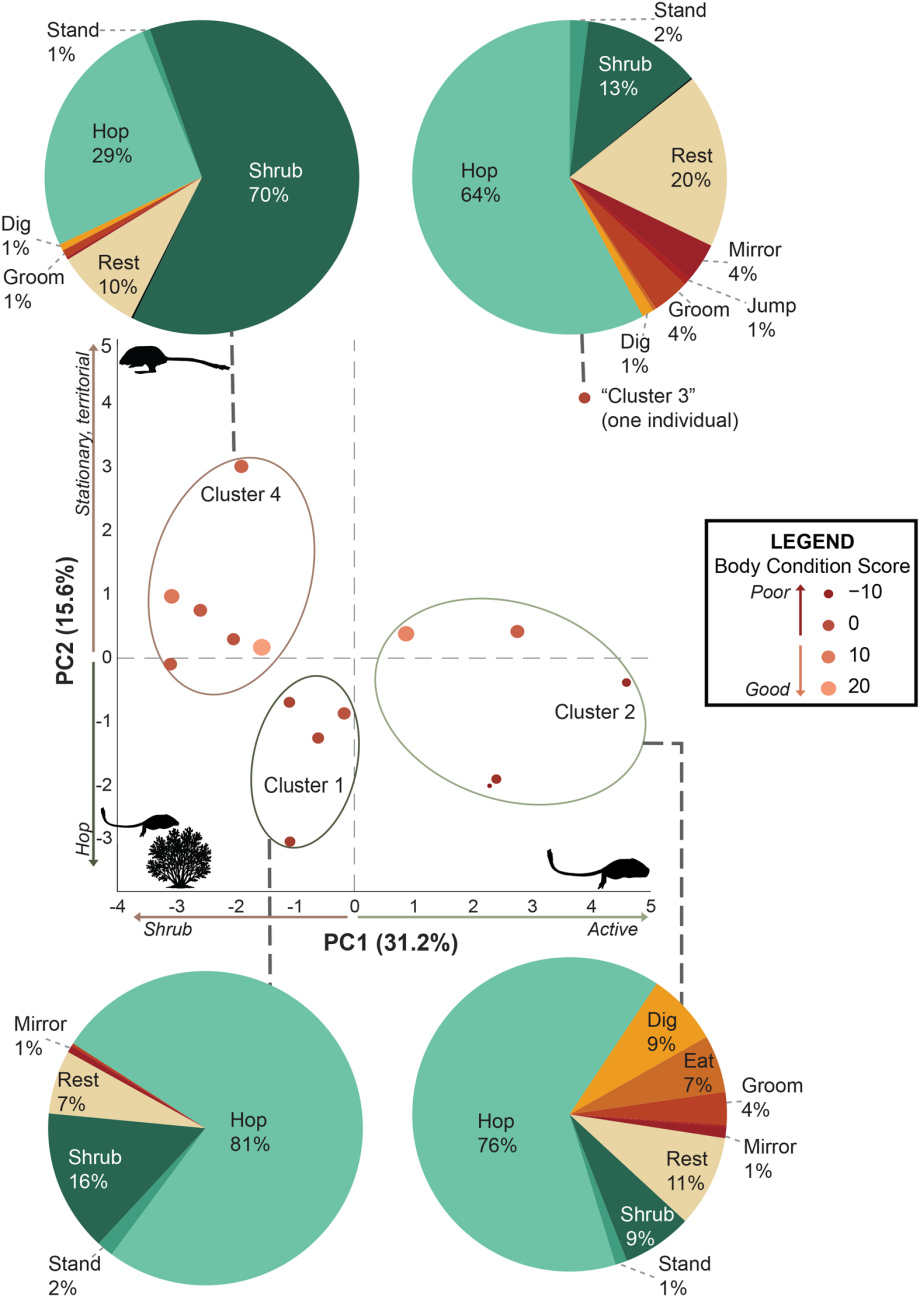
**Biplot of PC1 and PC2 depicting four clusters in observed behavior during the open arena test.** Pie charts associated with each cluster illustrate the average percent of time spent performing each behavior during the 15-min trial. Individuals in Cluster 1 with low PC1 and low PC2 scores spent the overwhelming majority (81%) of their time locomoting around the arena with some time spent in the shrub and resting and little time spent standing and interacting with the mirror. Individuals in cluster 2 had high PC1 scores but low PC2 scores and also spent most (76%) of their time locomoting but the other 24% of the time, they performed a variety of behaviors including digging and eating, grooming, interacting with the mirror, resting, standing, and hiding in the shrub. Cluster 3 consists of only one individual who is unique in that he spent the majority (64%) of his time locomoting but also spent a lot of time (20%) of time resting in the open, some (13%) time in the shrub, and little time devoted to other behaviors. Lastly, individuals in cluster 4 had low PC1 scores but high PC2 scores and spent most (70%) of the trial in the shrub, 29% of the time locomoting, some time spent resting in the open, and little time digging, grooming, and standing.

#### Predictors of locomotor measures

##### Mean speed

**Fig. 6.**
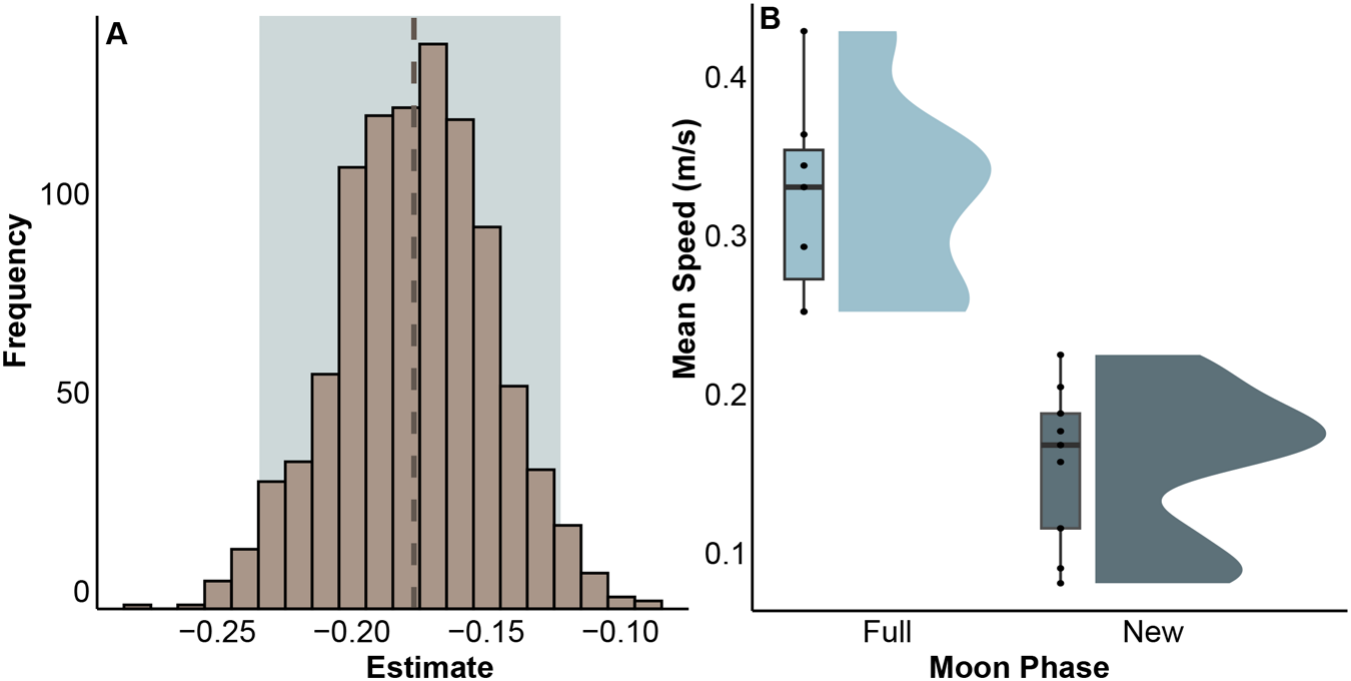
**(A) Bootstrapped estimate (coefficient) for mean speed (m/s) and moon phase.** The dashed line indicates the mean, and the shaded box indicates the 95% confidence interval. (B) Distribution plots of mean speed (m/s) during full (light blue) and new (dark blue) moon phases.

The model of best fit for predicting mean locomotor speed included moon phase where kangaroo rats used faster mean speeds during full moon phase compared to the new moon phase (*P*<0.001, Table 1 and Table S4, Fig. 6). However, because our sample reflected a bi-modal distribution of moon phases rather than systematic sampling across the full lunar cycle, further testing is warranted to more fully evaluate the role of moonlight in shaping locomotor behavior.

##### Total distance traveled

**Fig. 7.**
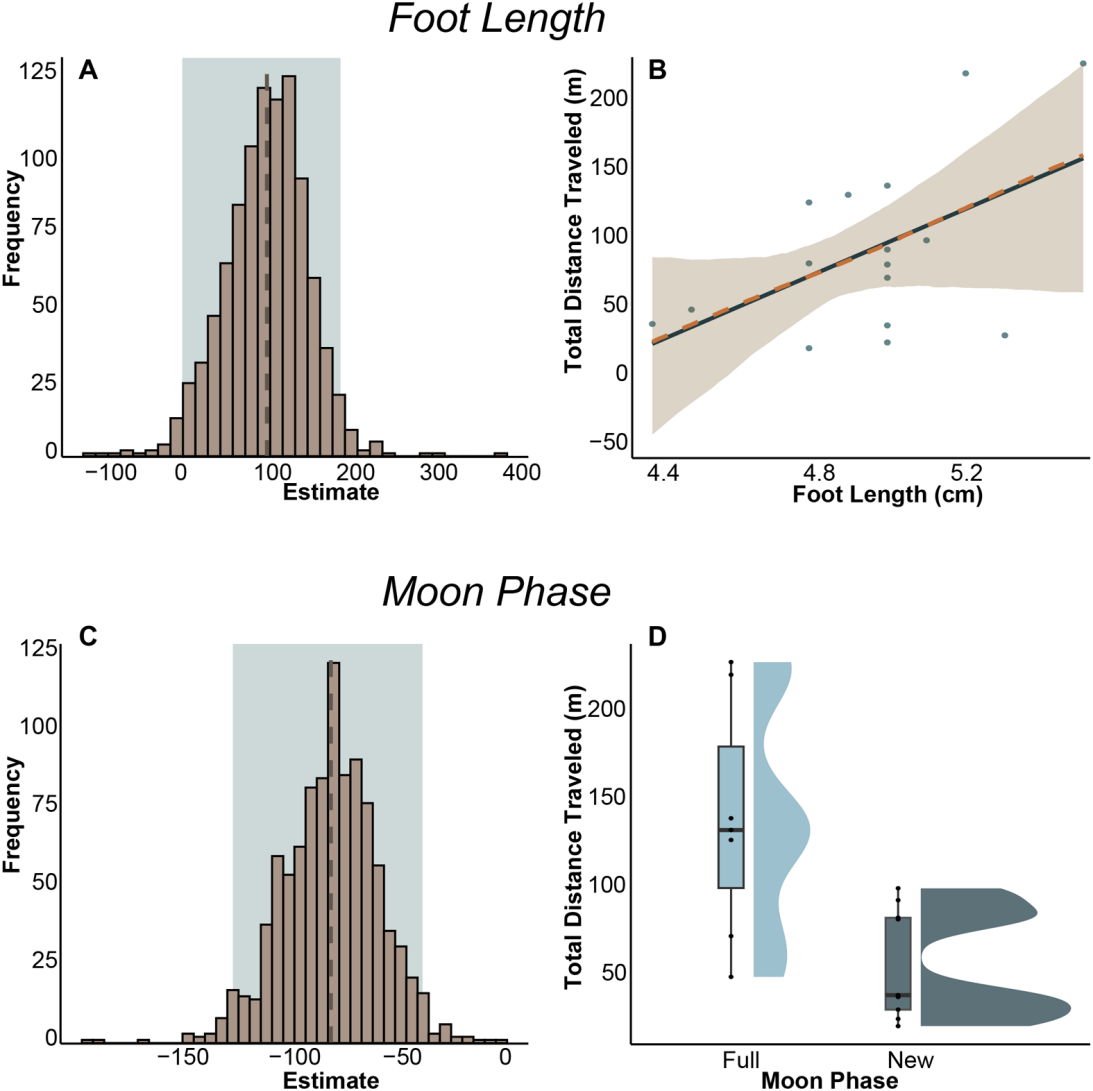
**(A) Bootstrapped estimates (slope) of regression between distance traveled (m) and foot length (cm).** The dashed line indicates the mean, and the shaded box indicates the 95% confidence interval. Notably, the mean slope exceeds 100, which would be expected under isometric scaling alone. (B) Bootstrapped regression of distance traveled and foot length where the solid line represents the slope of the original model (R^2^=0.51), the dashed line represents the mean of the bootstrap results, the ribbon indicates the 95% confidence interval, and the points indicate the original data. (C) Bootstrapped estimate (coefficient) for distance traveled (m) and moon phase. The dashed line indicates the mean, and the shaded box indicates the 95% confidence interval. (D) Distribution plots of distance traveled (m) during full (light blue) and new (dark blue) moon phases.

The best-fit model for predicting total distance traveled included moon phase and foot length. Kangaroo rats traveled farther during trials on full moon nights compared to new moon nights (*P*=0.002), and individuals with longer foot lengths also traveled greater distances over the 15-min trial (Table 1 and Table S4, Fig. 7).

Although the model result for foot length was statistically significant (*P*=0.03), the bootstrapped results revealed inconsistencies. The mean bootstrapped slope was slightly greater than the actual model's estimate (bootstrapped: 120.30; actual: 102.95) and the bootstrapped confidence interval overlapped zero (−14.29, 239.61), suggesting that variation in the dataset and small sample size limit the reliability of this result.

Additionally, since foot length did not influence mean speed, this suggests that larger individuals spend more time moving rather than moving faster. However, foot length had no significant effect on PC1, which captured variation between active and hiding behavior. To further explore this relationship, we performed a linear regression predicting PC1 from foot length, which approached significance (estimate=0.44, *P*=0.08). However, due to large variation in the dataset, bootstrap estimates and confidence intervals were unreliable and thus are not reported further.

### Correlation analysis

We also evaluated the correlation between the first two PCs and the locomotor measures (total distance traveled, mean speed). We found no significant relationships among the variables, though the association between PC1 and total distance traveled approached significance (*P*=0.11; Table S5).

## DISCUSSION

Our study reveals that desert kangaroo rats exhibit individual variation in behavioral responses when exposed to an open field test, and some of this variation can be attributed to morphological factors including skeletal proportions, sex, relative body condition, as well as moon phase. However, our data did not completely support our hypothesis that larger individuals exhibit more exploratory behaviors and faster speeds. Instead, we found that (1) individuals with poorer body condition had higher PC1 scores, indicating more time spent outside the shrub (Fig. 3); (2) males were more exploratory than females (Fig. 3); (3) individuals with poorer body condition and those active during full moon nights spent proportionally more time hopping rather than resting when outside the shrub, as reflected in lower PC2 scores (Fig. 4); and (4) individuals with larger feet traveled greater distances within the behavioral arena (Fig. 7). These results are consistent with previous work that found that zebra finches (*Taeniopygia guttata*) and yellow-bellied marmots (*Marmota flaviventer*) with poorer body conditions were more exploratory (Crino et al., 2017; Petelle et al., 2019). Exploratory behavior in Belding's ground squirrels (*Urocitellus beldingi*) did not differ between individuals with varying body conditions (Dosmann et al., 2015). Thus, the relationship between body condition and exploration may be context-dependent and vary across species, space, and time. In our study, healthier, larger individuals may have prioritized safety over food acquisition in the novel environment, remaining under shrub cover because their lower immediate need for foraging allowed them to prioritize avoidance of predator exposure. While *D. deserti* often initially escape into the open, and then to a burrow, the absence of a nearby burrow may lead them to seek the cover of a shrub (personal observation, C.M.). Because our analysis does not account for the sequence in a trial when specific behaviors occurred, our findings reflect overall tendencies across the trial rather than initial escape responses. In this context, risk-avoidant individuals may have remained under cover to prioritize safety, while individuals in poorer condition accepted greater risk exposure to forage and meet energetic demands.

Similarly, differences in exploratory behavior between sexes appears to be species dependent. In dark-eyed juncos (*Junco hyemalis*), males exhibit greater exploratory behavior, while female fawn-footed mosaic-tailed rats (*Melomys cervinipes*) are more exploratory (Atwell et al., 2012; Delarue et al., 2020). In contrast, there is no difference in exploratory behavior between sexes in black-capped chickadees (*Poecile atricapillus*; Thompson et al., 2018). In our study, we found that males were more exploratory and spent more time outside of the shrub when compared to females (as indicated by PC1 scores; Table S4). Sex-specific behaviors vary among different members of *Dipodomys*. For instance, males of *D. merriami* exhibit heightened aggression (Newmark and Jenkins, 2000), whereas in *D. heermanni*, dominance appears unaffected by sex (Shier and Randall, 2007). Prior work on *D. deserti* found no difference in response to predation risk or conspecific chasing behavior between the two sexes (Randall and Boltas King, 2001; Sullivan, 2000). While it is suggested that the *D. deserti* breeding season does not begin until January (Best et al., 1989), most data on this species' reproductive habits comes from laboratory observations and thus, their natural breeding behavior may differ or even occur year-round. During our second field season in December 2022, we trapped three juveniles, which (according to their weight, 55-92 gm) would have only been 1-2 months old (Butterworth, 1961), meaning they were born in October/November and conceived a month prior. (Note: these juveniles were not included in the behavioral analysis of this study due to the confounding factor of age.) One possible explanation for the difference in exploratory behavior between males and females may be the males' pursuit of opportunistic breeding. In banner-tailed kangaroo rats (*D. spectabilis*), males actively search and sometimes compete for access to females (Randall, 1991). Although this behavior has not been studied in *D. deserti*, it is conceivable that males of this species similarly seek out females, potentially explaining their increased exploratory activity within the arena. It is also possible that sex differences in territoriality contribute to these patterns; however, data are not available to provide a definitive mechanistic interpretation.

Individuals with higher body condition scores exhibited less exploratory behavior and displayed a greater inclination towards territorial activities such as sandbathing and interacting with the mirror (Fig. 5). Sandbathing is a behavior where kangaroo rats roll in the sand to deposit oils and scent-mark the area and has been associated with dominance in the banner-tailed kangaroo rat (Randall, 1987). Moreover, since kangaroo rats likely lack self-recognition, interacting with the mirror suggests confrontational conspecific behavior. Thus, individuals in better body condition – who may have more to lose and therefore act more cautiously – did not risk leaving the shrub to explore and forage in exposed areas and instead prioritized territorial behaviors. This pattern likely reflects a trade-off between food acquisition and safety, consistent with broader principles of risk management. Similar findings have been documented in various species such as the Galápagos hawk (*Buteo galapagoensis*), Eurasian nuthatches (*Sitta europaea*), and great tits (*P. major*) where individuals with poorer body condition scores were also less territorial (Diatroptov and Opaev, 2023; Snijders et al., 2017; Whiteman and Parker, 2004). Since territorial individuals may have increased access to resources, it tracks that they are also less exploratory and showed less interest in foraging.

Although we did not originally design our study to examine the effects of moon phase, we considered the potential for this effect to contribute to observed behavioral variation because of the contrast between the new moon during our 2021 field season and the full moon during 2022. We found that desert kangaroo rats used faster speeds and traveled greater distances on full moon nights compared to new moon nights. The increase in speed may represent an antipredator strategy, enhancing the likelihood of successful escape when predators have greater visual advantage under brighter conditions. However, the increase in distance traveled is more difficult to interpret, as it contrasts with patterns observed in *D. merriami*, where individuals that traveled farther were more likely to be depredated (Daly et al., 1990). In the context of our open field tests that prevented immediate escape into burrows, longer distances traveled may reflect efforts to search for an escape from the arena to return to the safety of burrows. Additionally, considering the larger body size of *D. deserti*, compared to *D. merriami*, they may face somewhat reduced predation risk and be able to travel farther before retreating underground. Notably, previous studies have reported limited effects of lunar illumination on heteromyid rodents: for instance, *D. merriami* did not alter resting, foraging, or movement behaviors across moon phases (Hanscom et al., 2023), and *D. deserti* did not shift foraging in open habitats under artificial illumination (Kotler, 1984). Together, our findings suggest that while moonlight may not broadly suppress activity, it can shape aspects of movement strategy such as speed and distance traveled in specific contexts, potentially reflecting species-specific trade-offs between foraging opportunities and predation risk. However, because our observations were based on an opportunistic, bi-modal distribution of moon phase conditions rather than systematic sampling across the full lunar cycle, further investigation with more comprehensive sampling is needed to confirm these patterns.

We used foot length of the kangaroo rat as an index of an individual's overall skeletal measurements. Since we did not use chemical restraint in our experiment, foot length is the most accurate measurement we could obtain (compared to skull or body length) while minimizing the stress to the animal. Assuming the foot length scales isometrically with other skeletal elements, this can be used to assess how body size may influence behavior. We found that larger individuals traveled longer distances during the 15-min trial (Table 1, Fig. 7). This pattern may have been driven in part by the largest individual in our sample, which could be considered an outlier, but we chose to retain this individual in the analysis and addressed the potential skew by bootstrapping the model. However, contrary to our hypothesis, larger individuals did not exhibit higher levels of exploratory behavior; rather, their ability to cover larger distances in the same time frame as smaller individuals was attributed to their longer limbs and stride lengths. This may reflect a general locomotor advantage among larger individuals, allowing them to traverse greater distances and reducing their exposure to predators in open habitats. By covering more ground during a given foraging bout, they may also have greater access to dispersed food resources compared to their smaller counterparts, who may need to spend more time in exposed areas to obtain the same amount of food.

While other species have been studied in the field using various techniques (i.e. Aliperti et al., 2021; Ovadia et al., 2005), this study was the first to analyze desert kangaroo rat behavior within their natural habitat using an open field test. Our primary objective was to measure individual variation in behavior in a controlled open-field test that exposed individuals to a consistent novel scenario, while keeping them within their natural environmental conditions, to understand natural behavioral variation within *D. deserti*. Captive animals in laboratory conditions may change their behavior for multiple reasons, due to responses to a consistent food supply, frequent encounters with humans, and lack of predation risk, which could influence behavioral responses to open field tests in laboratory conditions. However, there are trade-offs with this experimental design choice, and several limitations must be acknowledged. The traps themselves were novel objects, potentially attracting bolder individuals, while the use of sunflower seeds as bait may have enticed risk-averse but hungry animals. These factors may have biased sampling of the population and influenced foraging dynamics within the arena. We also could not control how long each individual remained in the trap as traps were set 1-2 h before sunset and checked 1-2 h after sunset, introducing variability in pre-trial stress. Although all trials took place between 10PM and 2AM, exploratory behavior may vary with time of night. Human presence and handling may have also influenced behavior despite efforts to minimize stress by using low voices and limiting movement. Environmental variables like cloud cover and temperature, could also have influenced behavior. Additionally, we were unable to account for life history traits such as prior experience, reproductive status, parasite load, or hunger level. Lastly, although our trapping effort was substantial and yielded a sample of 16 adult individuals, this remains a relatively limited dataset from a single population exposed to a particular set of resources and risks, and behaviors could differ in other populations facing different ecological conditions. Future studies would benefit from replicating this work with larger samples and across multiple populations of *D. deserti* to more fully capture variation within the species. In addition, our observations of moon phase warrant further investigation with more systematic sampling across the lunar cycle, as our data reflected a bimodal distribution of moon phase conditions rather than a balanced design. Nonetheless, despite these constraints, limited data exists in the literature on *D. deserti*, and therefore our study provides useful groundwork in suggesting how individual characteristics may interact with environmental factors to influence behavioral patterns in desert kangaroo rats.

We found that exploratory behavior in kangaroo rats was influenced by sex and body condition while territorial behavior (as represented by PC2) was influenced only by body condition (Fig. 4 and 5). Interestingly, contrary to previous findings in other species (Agnani et al., 2020; Aliperti et al., 2021; Schirmer et al., 2019), we did not uncover correlations between behavioral suites and measures of locomotor performance (e.g. movement speed). This suggests that, in this species, exploratory or risk-averse behaviors may not be directly tied to locomotor capabilities. It is possible that individual variation supports niche differentiation without requiring a strong behavioral-locomotor link. Alternatively, our study design may have limited our ability to detect such relationships, and future work should further evaluate how desert kangaroo rat behavior may be altered by variables such as distance to cover, time of night, moon phase, season, and weather. In particular, follow-up studies using longer observation periods with systematic sampling and experimental control of key predictors identified here would provide a stronger test of these factors. Additionally, incorporating repeated trials or assessments of maximum and preferred speeds may better capture the nuances of this potential connection. Moreover, our divergent results underscore the necessity of examining such patterns across a broader spectrum of species. This need is particularly acute for *D. deserti*, a species that has been rarely studied in the field, meaning that even baseline information on their behavior in natural contexts is limited. Our findings therefore provide an important step toward filling this knowledge gap and suggesting specific hypotheses to test in future field work studies. Understanding the predictors of variation in species behavior, such as habitat characteristics, anthropogenic influences, and predator and conspecific densities, is crucial for understanding movement ecology. Furthermore, this study highlights the necessity for more comprehensive behavioral research on *D. deserti* in areas such as reproductive behavior in natural settings, male-female interactions and behavioral disparities, and the impact of body condition on overall fitness. Given the ecological significance of *D. deserti* within desert ecosystems, a deeper understanding of their behavioral ecology is imperative as this could impact future habitat restoration efforts, especially if males and females differ in how they navigate the trade-off between acquiring food in exposed areas and maintaining safety under shrub cover, as suggested by this research. Continued knowledge of wildlife behavior not only enhances our comprehension of ecosystem functioning but also lays the groundwork for informed conservation strategies, ensuring the preservation of this species and the integrity of its habitat for future generations.

## MATERIALS AND METHODS

### Field site

We respectfully acknowledge that this work took place on the traditional land of the Moapa Band of Paiute Indians in the Mojave Desert in Nevada, USA, nearing the Arizona border. This work was approved by USC IACUC (protocol #21394) and conducted on permits issued by the Nevada Department of Wildlife and the Bureau of Land Management (license # 40701). The landscape at this site consists of mountains and rock formations, and soft sand dunes: the preferred habitat of the burrowing *D. deserti*. We constructed a behavioral arena for the open field test (Fig. 1) in a location central to many identified small mammal burrows. This arena was an enclosure made of garden cloth and stakes, measuring 3 m in diameter. To encourage a wider range of behaviors in the arena, we included a shrub (approximately 0.5-m diameter) near the wall, a mirror placed approximately 1 m away from the shrub along the wall, and a centrally placed food patch, in view of the mirror. The food patch was seeded with a handful of millet seed before the start of each trial, and the seed was lightly covered with sand. We leveled out the substrate between trials; however, this may not have been sufficient to remove all signs and scents from previous individuals. Five IR-modified GoPro cameras were placed around the arena and infrared lights were used to illuminate the arena during behavior trials.

### Data collection

Kangaroo rats were captured in extra-long Sherman traps baited with sunflower seeds that were set just before sunset and were checked 1 h after sunset. Trapped individuals were transported to the behavior arena, which was typically not more than ∼200 m away from the trapping site. We recorded the sex, mass (with hanging spring scale), and foot length prior to behavior trials. Individuals were PIT tagged (a small microchip inseed between the shoulder blades) to ensure future identification in case of recapture on subsequent nights.

We collected data in the winter of two consecutive years: October 2021 and December 2022. In 2021, we trapped a total of nine *D. deserti* (five females, four males). In 2022, we trapped a total of 11 *D. deserti* (three females, eight males), but three of the individuals (two males, one female) were juveniles and were not used in the analysis to avoid the potential confounding factor of age. Additionally, we recaptured one male in 2022 that we had already tested in 2021 (identified via his PIT tag) and thus we did not use his 2022 trial in our analysis. Notably, the moon was in different phases during our two field collection trips – new moon in 2021 and full moon in 2022. Although this difference was unintentional and not considered when planning field dates, we included moon phase as a factor in our statistical analyses to account for potential behavioral differences between these lunar phases.

After taking morphological measurements (Table S1), we began behavioral trials by conducting a modified open field behavioral test. All lights (apart from the IR lights) were turned off, cameras were turned on, and an identified individual was placed in the arena. At the time of the animals' contact with the ground, we started a 15-min timer, and all researchers stepped away from the arena for the duration of the behavior trial. The arena was reset after each trial (if the food patch was disturbed) and we then began the next trial until all captured animals for the night had been observed. After behavior recordings, some kangaroo rats were released back into the environment after the trial, and some were transported to USC for unrelated experiments by C.M.

### Behavioral analysis

We created an ethogram and tracked a total of ten behaviors: dig, eat, groom, jump, mirror interaction, rest, sandbathe, hiding in shrub, stand, and hop (Table S2) using the annotation software *Elan* (ELAN, 2022, Sloetjes and Wittenburg, 2008). Using this program, we tagged each behavior over the 15-min trial from each camera view, then synced and merged the tracked files together for a complete timeline of behaviors performed during the trial. We used the Elan Toolbox in Matlab (Spiro and Himberg, 2016) to calculate summary values (number of bouts and total time) of the behaviors. Lastly, we converted total time to the proportion of time an individual spent on each behavior during the trial. We used proportion of time and number of bouts for each behavior in a principal component analysis to evaluate correlated behaviors.

### Locomotion analysis

We placed five calibration stakes in the arena and measured the distance between them to use for 2D video calibration. The stakes remained in the arena for the duration of the trial. Due to logistical constraints, we did not employ 3D tracking methods, as our stake setup proved unsuitable for reliable 3D calibration. Additionally, while an overhead camera could have provided supplementary data, its necessity was not initially anticipated and thus we collected video data using just the five cameras surrounding the arena. We calibrated our videos with the 2D information and tracked all instances of locomoting behavior throughout the trial using Kinovea (Charmant, 2023). For each locomotor bout, we selected the camera that provided the clearest lateral view of the animal, ensuring that its movement remained fully in frame. This approach allowed us to track 2D motion consistently across all individuals, as locomotor metrics were always derived from a single clear lateral view. From these tracked positions, we calculated the average speed and distance traveled per bout of locomotion.

### Statistical analysis

We calculated relative body condition by fitting a linear relationship between body mass (g) and foot length (cm) using least squares regression, obtaining the residuals from this relationship and then used the residual values as the individual's relative body condition score (Schulte-Hostedde et al., 2005). This analysis assumes that the skeletal measure of foot length represents the animal's skeletal dimensions, and that body mass may vary depending on an individual's soft-tissue mass relative to skeletal size. Residuals above the line indicate animals with high body mass relative to skeletal dimensions (high body conditions), and residuals below the line indicate animals with low body mass relative to skeletal dimensions (low body condition).

We used a principal component analysis to evaluate correlations among tracked behaviors and examine the main sources of behavioral variation in the dataset. We then ran two linear mixed-effects models to test for predictors of (1) the first two principal component scores (which explained 31.2% and 15.6% of the variance, respectively) and (2) locomotor variables (average speed and total distance traveled). Fixed effects included anatomical variables (sex, relative body condition, and foot length), moon phase, and trial order – the sequence of each kangaroo rat during data collection (1-6), included to account for potential behavioral effects of scent cues or other signs left by previous individuals. The day of the trial was included as a random effect. We performed model selection to identify the model of best fit for the first two PCs and for both locomotor measures. The model with the lowest AIC score was determined to be the model of best fit and if that model included more than one fixed effect, we compared the original model to a model including interaction terms between the response variables and kept the model with the lowest AIC score.

Some coefficient estimates from the linear mixed effects model were close to zero, prompting us to perform a bootstrap of these estimates using the ‘boot’ package in R (R Core Team, 2021) with 1000 replicates to obtain more reliable uncertainty measures. Additionally, we computed 95% confidence intervals of the estimates from this analysis to better understand and visualize the variation in our results. To remain consistent in our analysis, we applied this bootstrap approach across all models, regardless of their initial magnitude or statistical significance.

### Artificial intelligence tools

We acknowledge the use of OpenAI's ChatGPT (GPT-5) to assist with editing sentence structure, clarifying language, and drafting code for figure preparation. All content was subsequently reviewed and verified by the authors.

## Supplementary Material

10.1242/biolopen.062164_sup1 Supplementary information

## References

[BIO062164C1] Agnani, P., Thomson, J., Schradin, C. and Careau, V. (2020). The fast and the curious II: Performance, personality, and metabolism in Karoo bush rats. *Behav. Ecol. Sociobiol.* 74, 123. 10.1007/s00265-020-02908-y

[BIO062164C2] Aliperti, J. R., Davis, B. E., Fangue, N. A., Todgham, A. E. and Van Vuren, D. H. (2021). Bridging animal personality with space use and resource use in a free-ranging population of an asocial ground squirrel. *Anim. Behav.* 180, S000334722100244X. 10.1016/j.anbehav.2021.07.019

[BIO062164C3] Atwell, J. W., Cardoso, G. C., Whittaker, D. J., Campbell-Nelson, S., Robertson, K. W. and Ketterson, E. D. (2012). Boldness behavior and stress physiology in a novel urban environment suggest rapid correlated evolutionary adaptation. *Behav. Ecol.* 23, 960-969. 10.1093/beheco/ars059 22936840 PMC3431113

[BIO062164C4] Best, T. L., Hildreth, N. J. and Jones, C. (1989). Dipodomys deserti. *Mamm. Species* 339, 1-8. 10.2307/3504260

[BIO062164C5] Brown, C., Jones, F. and Braithwaite, V. A. (2007). Correlation between boldness and body mass in natural populations of the poeciliid Brachyrhaphis episcopi. *J. Fish Biol.* 71, 1590-1601. 10.1111/j.1095-8649.2007.01627.x

[BIO062164C6] Butterworth, B. B. (1961). The breeding of *Dipodomys deserti* in the laboratory. *J. Mammal.* 42, 413-414. 10.2307/1377057

[BIO062164C7] Charmant, J. (2023). *Kinovea* (Version 0.9.5) [Computer software].

[BIO062164C8] Christensen, B. A., Lin, D. C., Schwaner, M. J. and McGowan, C. P. (2022). Elastic energy storage across speeds during steady-state hopping of desert kangaroo rats (*Dipodomys deserti*). *J. Exp. Biol.* 225, jeb242954. 10.1242/jeb.242954 35019972

[BIO062164C9] Crino, O. L., Buchanan, K. L., Trompf, L., Mainwaring, M. C. and Griffith, S. C. (2017). Stress reactivity, condition, and foraging behavior in zebra finches: Effects on boldness, exploration, and sociality. *Gen. Comp. Endocrinol.* 244, 101-107. 10.1016/j.ygcen.2016.01.014 26828818

[BIO062164C10] Daly, M., Wilson, M., Behrends, P. R. and Jacobs, L. F. (1990). Characteristics of kangaroo rats, *Dipodomys merriami*, associated with differential predation risk. *Anim. Behav.* 40, 380-389. 10.1016/S0003-3472(05)80934-0

[BIO062164C11] Delarue, E. M. P., Kerr, S. E. and Rymer, T. L. (2020). Habitat and sex effects on behaviour in fawn-footed mosaic-tailed rats (Melomys cervinipes). *Aust. Mammal.* 43, 319-329. 10.1071/AM19065

[BIO062164C12] Diatroptov, M. and Opaev, A. (2023). Bigger male Eurasian nuthatches (Sitta europaea) behave more aggressively in playback-simulated territorial intrusion. *J. Ethol.* 41, 185-193. 10.1007/s10164-023-00784-3

[BIO062164C13] Dosmann, A. J., Brooks, K. C. and Mateo, J. M. (2015). Within-individual correlations reveal link between a behavioral syndrome, condition, and cortisol in free-ranging Belding's ground squirrels. *Ethology* 121, 125-134. 10.1111/eth.12320 25598565 PMC4295653

[BIO062164C14] Eilam, D. (2005). Die hard: A blend of freezing and fleeing as a dynamic defense—implications for the control of defensive behavior. *Neurosci. Biobehav. Rev.* 29, 1181-1191. 10.1016/j.neubiorev.2005.03.027 16085311

[BIO062164C15] ELAN (Version 6.3). (2022). [Computer software]. Max Planck Institute for Psycholinguistics, The Language Archive, Nijmegan, The Netherlands. https://archive.mpi.nl/tla/elan

[BIO062164C16] Freymiller, G. A., Whitford, M. D., Higham, T. E. and Clark, R. W. (2019). Escape dynamics of free-ranging desert kangaroo rats (Rodentia: Heteromyidae) evading rattlesnake strikes. *Biol. J. Linn. Soc.* 127, 164-172. 10.1093/biolinnean/blz027

[BIO062164C17] Goldingay, R. L., Kelly, P. A. and Williams, D. F. (1997). The Kangaroo Rats of California: Endemism and conservation of keystone species. *Pac. Conserv. Biol.* 3, 47-60. 10.1071/PC970047

[BIO062164C18] Gould, T. D., Dao, D. T. and Kovacsics, C. E. (2009). The open field test. In *Mood and Anxiety Related Phenotypes in Mice: Characterization Using Behavioral Tests* (ed. T. D. Gould), pp. 1-20. Humana Press.

[BIO062164C19] Hanscom, R. J., Hill, J. L., Patterson, C., Marbach, T., Sukumaran, J., Higham, T. E. and Clark, R. W. (2023). Cryptic behavior and activity cycles of a small mammal keystone species revealed through accelerometry: a case study of Merriam's kangaroo rats (Dipodomys merriami). *Mov. Ecol.* 11, 72. 10.1186/s40462-023-00433-x 37919756 PMC10621205

[BIO062164C20] Harris, S., Ramnarine, I. W., Smith, H. G. and Pettersson, L. B. (2010). Picking personalities apart: estimating the influence of predation, sex and body size on boldness in the guppy Poecilia reticulata. *Oikos* 119, 1711-1718. 10.1111/j.1600-0706.2010.18028.x

[BIO062164C21] Hughes, B., Bowman, J. and Schulte-Hostedde, A. (2024). Exploratory and risk-taking behaviours in coexisting rodents*.* *bioRxiv* 2024.01.09.574853. 10.1101/2024.01.09.574853

[BIO062164C22] Kelleher, S. R., Silla, A. J., Dingemanse, N. J. and Byrne, P. G. (2017). Body size predicts between-individual differences in exploration behaviour in the southern corroboree frog. *Anim. Behav.* 129, 161-170. 10.1016/j.anbehav.2017.05.013

[BIO062164C23] Kern, E. M. A., Robinson, D., Gass, E., Godwin, J. and Langerhans, R. B. (2016). Correlated evolution of personality, morphology and performance. *Anim. Behav.* 117, 79-86. 10.1016/j.anbehav.2016.04.007 29398712 PMC5791543

[BIO062164C24] Korsten, P., van Overveld, T., Adriaensen, F. and Matthysen, E. (2013). Genetic integration of local dispersal and exploratory behaviour in a wild bird. *Nat. Commun.* 4, 2362. 10.1038/ncomms3362 23974327

[BIO062164C25] Kotler, B. P. (1984). Risk of predation and the structure of desert rodent communities. *Ecology* 65, 689-701. 10.2307/1938041

[BIO062164C26] Lima, S. L. and Dill, L. M. (1990). Behavioral decisions made under the risk of predation: a review and prospectus. *Can. J. Zool.* 68, 619-824. 10.1139/z90-092

[BIO062164C27] Longland, W. S. and Ostoja, S. M. (2013). Ecosystem services from keystone species: diversionary seeding and seed-caching desert rodents can enhance indian ricegrass seedling establishment. *Restor. Ecol.* 21, 285-291. 10.1111/j.1526-100X.2012.00895.x

[BIO062164C28] López, P., Hawlena, D., Polo, V., Amo, L. and Martín, J. (2005). Sources of individual shy–bold variations in antipredator behaviour of male Iberian rock lizards. *Anim. Behav.* 69, 1-9. 10.1016/j.anbehav.2004.05.010

[BIO062164C29] McGowan, C. P. and Collins, C. E. (2018). Why do mammals hop? Understanding the ecology, biomechanics and evolution of bipedal hopping. *J. Exp. Biol.* 221, jeb161661. 10.1242/jeb.161661 29907573

[BIO062164C30] Money, D. A., Ingley, S. J. and Johnson, J. B. (2017). Divergent predation environment between two sister species of livebearing fishes (Cyprinodontiformes: Poeciliidae) predicts boldness, activity, and exploration behavior. *Rev. Biol. Trop.* 65, 267-277. 10.15517/rbt.v65i1.23861 29466643

[BIO062164C31] Newmark, J. E. and Jenkins, S. H. (2000). Sex differences in agonistic behavior of Merriam's kangaroo rats (Dipodomys merriami). *Am. Midl. Naturalist* 143, 377-388. 10.1674/0003-0031(2000)143[0377:SDIABO]2.0.CO;2

[BIO062164C32] Ovadia, O., Abramsky, Z., Kotler, B. P. and Pinshow, B. (2005). Inter-specific competitors reduce inter-gender competition in Negev Desert gerbils. *Oecologia* 142, 480-488. 10.1007/s00442-004-1726-9 15655694

[BIO062164C33] Petelle, M. B., Martin, J. G. A. and Blumstein, D. T. (2019). Mixed support for state maintaining risky personality traits in yellow-bellied marmots. *Anim. Behav.* 150, 177-188. 10.1016/j.anbehav.2019.02.008

[BIO062164C34] R Core Team (2021). *R: A Language and Environment for Statistical Computing*. [Computer software]. R Foundation for Statistical Computing. Vienna, Austria, https://www.R-project.org/.

[BIO062164C35] Randall, J. A. (1987). Sandbathing as a territorial scent-mark in the bannertail kangaroo rat, Dipodomys spectabilis. *Anim. Behav.* 35, 426-434. 10.1016/S0003-3472(87)80267-1

[BIO062164C36] Randall, J. A. (1991). Mating strategies of a nocturnal, desert rodent (Dipodomys spectabilis). *Behav. Ecol. Sociobiol.* 28, 215-220. 10.1007/BF00172173

[BIO062164C37] Randall, J. A. and Boltas King, D. K. (2001). Assessment and defence of solitary kangaroo rats under risk of predation by snakes. *Anim. Behav.* 61, 579-587. 10.1006/anbe.2000.1643

[BIO062164C38] Rauber, R., Clutton-Brock, T. H. and Manser, M. B. (2019). Drought decreases cooperative sentinel behavior and affects vocal coordination in meerkats. *Behav. Ecol.* 30, 1558-1566. 10.1093/beheco/arz112

[BIO062164C39] Schirmer, A., Herde, A., Eccard, J. A. and Dammhahn, M. (2019). Individuals in space: personality-dependent space use, movement and microhabitat use facilitate individual spatial niche specialization. *Oecologia* 189, 647-660. 10.1007/s00442-019-04365-5 30826867 PMC6418052

[BIO062164C40] Schulte-Hostedde, A. I., Zinner, B., Millar, J. S. and Hickling, G. J. (2005). Restitution of mass–size residuals: validating body condition indices. *Ecology* 86, 155-163. 10.1890/04-0232

[BIO062164C41] Shaw, A. K. (2020). Causes and consequences of individual variation in animal movement. *Mov. Ecol.* 8, 12. 10.1186/s40462-020-0197-x 32099656 PMC7027015

[BIO062164C42] Shier, D. M. and Randall, J. A. (2007). Use of different signaling modalities to communicate status by dominant and subordinate Heermann's kangaroo rats (Dipodomys heermanni). *Behav. Ecol. Sociobiol.* 61, 1023-1032. 10.1007/s00265-006-0335-5

[BIO062164C43] Sih, A., Bell, A. M., Johnson, J. C. and Ziemba, R. E. (2004). Behavioral syndromes: an integrative overview. *Q Rev. Biol.* 79, 241-277. 10.1086/422893 15529965

[BIO062164C51] Sloetjes, H. and , Wittenburg, P. (2008). Annotation by category - ELAN and ISO DCR. In: Proceedings of the 6th International Conference on Language Resources and Evaluation (LREC 2008).

[BIO062164C44] Snijders, L., van Oers, K. and Naguib, M. (2017). Sex-specific responses to territorial intrusions in a communication network: evidence from radio-tagged great tits. *Ecol. Evol.* 7, 918-927. 10.1002/ece3.2686 28168028 PMC5288255

[BIO062164C45] Spiro, N. and Himberg, T. (2016). Analysing change in music therapy interactions of children with communication difficulties. *Philos. Trans. R. Soc. B Biol. Sci.* 371, 20150374. 10.1098/rstb.2015.0374 PMC4843612 27069051

[BIO062164C46] St. Juliana, J. R., Kotler, B. P., Wielebnowski, N. and Cox, J. G. (2017). Stress as an adaptation I: Stress hormones are correlated with optimal foraging behaviour of gerbils under the risk of predation. *Evol. Ecol. Res*. 18, 571-585.

[BIO062164C47] Sullivan, M. C. (2000). *Social Biology of the Desert Kangaroo Rat (Dipodomys deserti) (Rodentia: Heteromyidae)*. San Francisco State University.

[BIO062164C48] Thompson, M. J., Evans, J. C., Parsons, S. and Morand-Ferron, J. (2018). Urbanization and individual differences in exploration and plasticity. *Behav. Ecol.* 29, 1415-1425. 10.1093/beheco/ary103

[BIO062164C49] Van Oers, K., Drent, P. J., De Goede, P. and Van Noordwijk, A. J. (2004). Realized heritability and repeatability of risk-taking behaviour in relation to avian personalities. *Proc. R. Soc. Lond. B Biol. Sci.* 271, 65-73. 10.1098/rspb.2003.2518 PMC1691563 15002773

[BIO062164C50] Whiteman, N. K. and Parker, P. G. (2004). Body condition and parasite load predict territory ownership in the galápagos hawk. *The Condor* 106, 915-921. 10.1093/condor/106.4.915

