# Supplementary material for: Beyond the burrow: Body condition and sex influence exploratory behavior in desert kangaroo rats (Dipodomys deserti)
Source: Biol Open. 2025 Oct 15;14(10):bio062164. doi: 10.1242/bio.062164 (PMC12570148; doi:10.1242/bio.062164)
Supplement: Supplementary information [file biolopen-14-062164-s1.pdf]

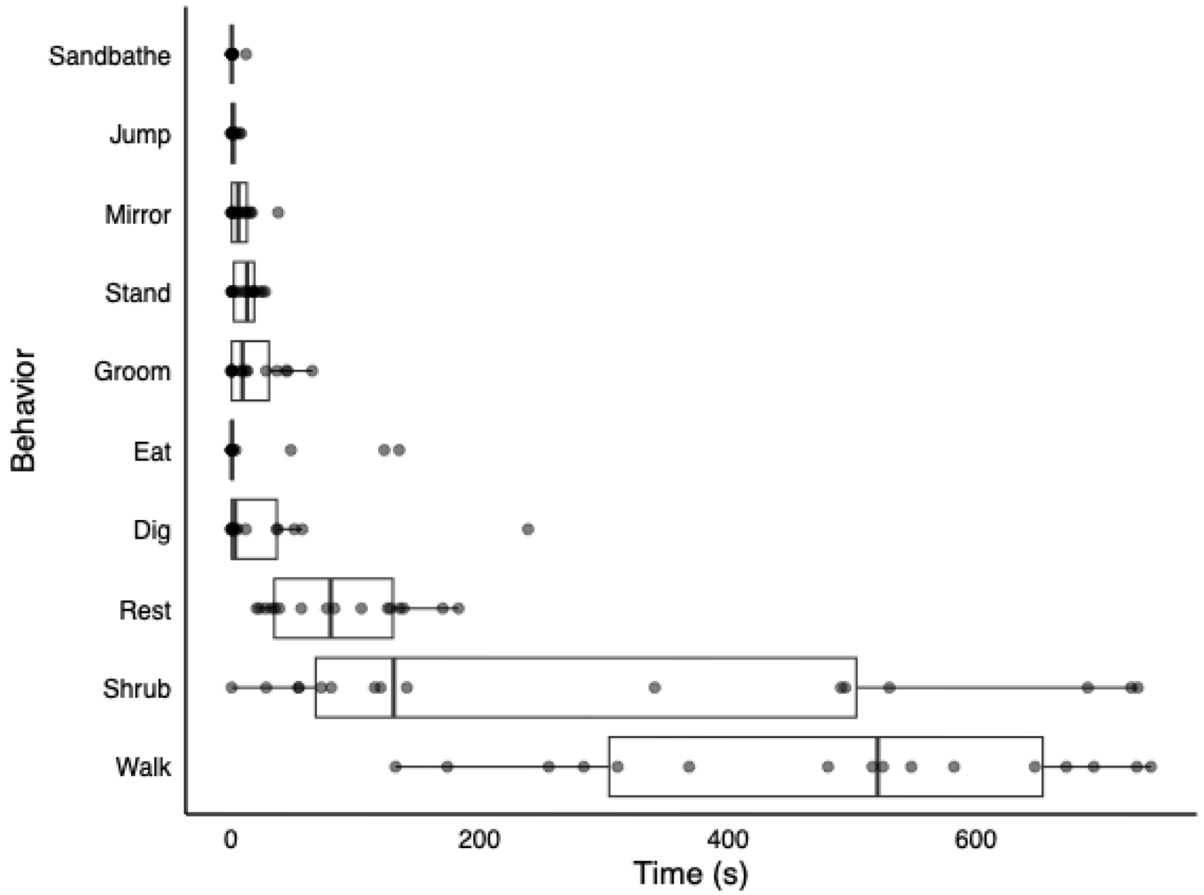

Fig. S1. Boxplots showing the variation in time allocation across different behavioral categories, organized from lowest to highest mean time spent (top to bottom).

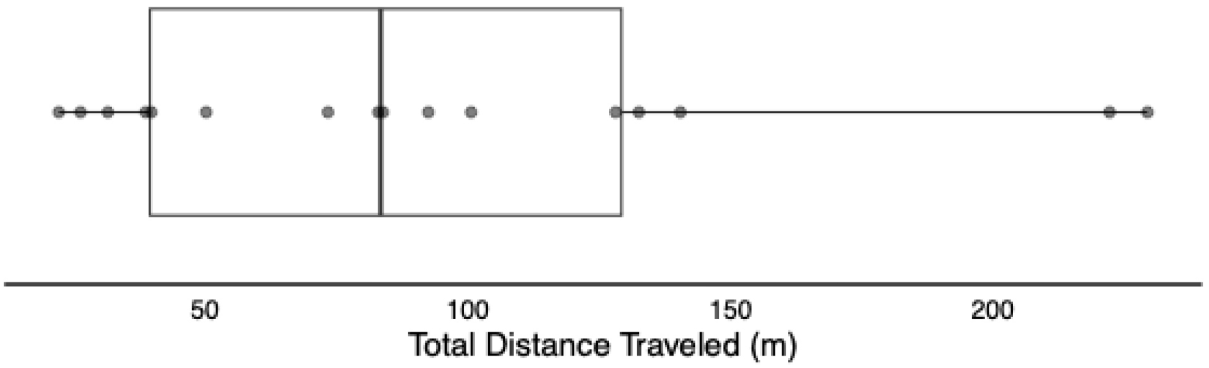

Fig. S2. Boxplot showing the range of total distance traveled (in meters) across all individuals (mean ± SD: 93.4 ± 64.2 m; range: 22.1–229.3 m).

Biology Open • Supplementary information

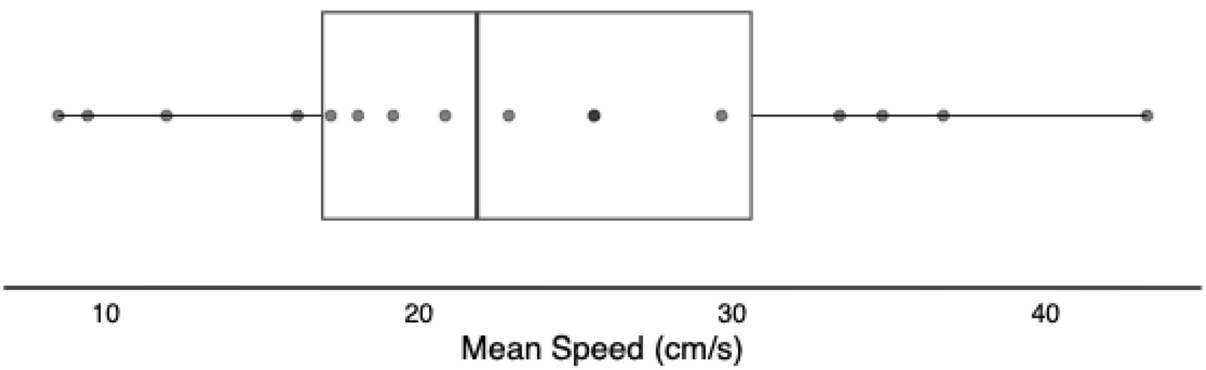

Fig. S3. Boxplot showing the range of average speed (in cm/s) across all individuals (mean ± SD: 23.3 ± 10.1 cm/s; range: 8.5–43.2 cm/s).

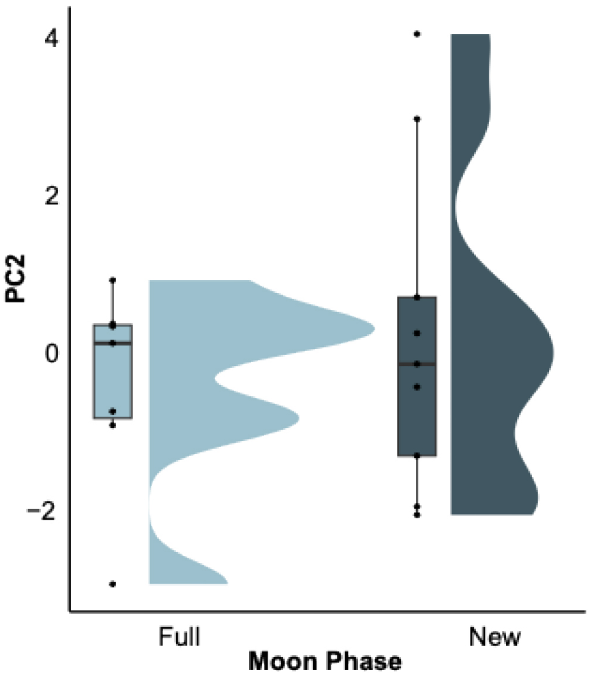

Fig. S4. Distribution plots of PC2 scores during full (light blue) and new (dark blue) moon phases.

Table S1. Data of trapped individuals from 2021 and 2022 seasons. Those shaded in gray were not used in the analysis either due to age class or previous capture.

	Name	*Number of Trials*	*Sex*	*Mass (gm)*	*Foot Length (cm)*
October 2021	FB_01	1	♀	100	5.1
	FB_02	2	♂	95	4.8
	FB_03	3	♀	85	4.4
	FB_04	1 (+2 in 2022)	♂	125	5.3
	FB_05	2	♀	100	4.8
	FB_06	1	♂	98	5
	FB_07	1	♀	115	5
	FB_08	1	♀	105	5
	FB_09	1	♂	90	5
December 2022	FB22_01	4	♂ (juvenile)	55	5
	FB_04	2 (+1 in 2021)	♂	135	5.1
	FB22_03	1	♂	100	4.5
	FB22_05	1	♂	138	5
	FB22_06	1	♀ (juvenile)	92	5
	FB22_07	1	♂	137	5.5
	FB22_08	1	♀	94	4.8
	FB22_09	1	♀	96	4.9
	FB22_10	1	♂ (juvenile)	58	4.9
	FB22_11	1	♂	137	5.2

Biology Open • Supplementary information

FB22_12	1	♂	110	5

Summary Data

Total Number of Individuals	*Total Number of Trials*	*Sex Ratio*	*Mass Range (gm)*	*Foot Length Range (cm)*
19	28	11 ♂ : 8 ♀	55-137 ($\bar{x}$=103)	4.4-5.5 ($\bar{x}$=4.9)

Table S2. Ethogram of 10 tracked behaviors during trials.

**Behavior**	**Definition**
Dig	Uses front legs to displace sand
Eat	Ingests food or stores food in cheeks (often associated with 'dig' behavior)
Groom	Uses front legs to rub face, licks fur, or licks tail
Jump	All four paws leave push off the ground and move vertically and/or horizontally
Mirror interaction	Looks at, climbs on, charges at, or explores mirror
Rest	Not moving location or performing other behaviors for >3 seconds
Sandbathe	Rolls on the sand, typically twisting the body
Shrub hiding	Individual is seen entering the shrub and remains hidden in the shrub
Stand	Maintain an upright position using only back legs
Hop	Individual hops or bounds through the arena without stopping for >3 seconds

Biology Open • Supplementary information

Table S3. Summary statistics (mean, standard deviation, and range) for time allocation and behavioral bout frequency across all observed behaviors, organized from lowest to highest mean time spent (top to bottom).

	**Time (seconds)**		**Number of Bouts**	
Behavior	***Mean ± SD***	***Range***	***Mean ± SD***	***Range***
Sandbathe	0.8 ± 2.9	0, 11.6	0.1 ± 0.3	0, 1
Jump	1.7 ± 2.3	0, 7.3	1.4 ± 1.8	0, 6
Mirror	8.0± 9.8	0, 37.5	2.3 ± 2.7	0, 9
Stand	11.6 ± 9.5	0, 27.0	8.1 ± 6.3	0, 19
Groom	16.7 ± 20.5	0, 64.9	1.6 ± 1.9	0, 6
Eat	19.3 ± 44.5	0, 134.9	0.8 ± 2	0, 8
Dig	27.6 ± 59.7	0, 238.7	2.8 ± 3.6	0, 11
Rest	86.0 ± 55.3	20.0, 182.7	9.4 ± 5.3	2, 23
Shrub	291.5 ± 273.5	0, 730.0	4.4 ± 2.5	0, 8
Hop	478.7 ± 200.7	132, 740.5	22 ± 10.1	8, 49

Table S4. Results of model selection based on AIC score.

Dependent Variable	Components of Best-fitting Model
PC1	*Sex * Body Condition Score + (1 | Date)*
PC2	*Body Condition Score + Moon Phase + (1 | Date)*
Mean Speed	*Moon Phase + (1 | Date)*
Total Distance Traveled	*Foot Length + Moon Phase + (1 | Date)*

Table S5. Correlation coefficient output for principal components and locomotor variables.

	Distance Traveled		*Speed*	
	R	*p-value*	*R*	*p-value*
PC1	0.42	0.11	0.08	0.78
PC2	-0.17	0.53	-0.06	0.83

Biology Open • Supplementary information
